# Economic Burden Analysis of Parkinson's Disease Patients in China

**DOI:** 10.1155/2017/8762939

**Published:** 2017-06-14

**Authors:** Jun-Xiu Yang, Lei Chen

**Affiliations:** Department of Neurology, Tianjin Union Medical Center, Tianjin 300121, China

## Abstract

**Background and Objective:**

Parkinson's Disease (PD) is a progressive neurodegenerative disorder, which is prevalent in people over 65 years old. PD reduces patients' quality of life and exerts a heavy economic burden on patients and their families. The purpose of this research is to identify the costs of PD and to evaluate the economic distribution of medical care for PD patients in China.

**Methods:**

A professional survey was administered to 116 patients with PD. Records of medical cost were reviewed. Direct and indirect costs were analyzed. The main cost-driving factors of PD were identified using multivariate regression analysis.

**Results:**

The average annual cost per PD patient in China is $3,225.94, with direct and indirect costs accounting for $2,503.46 and $722.48, respectively. Direct costs consist of $556.27 for surgery, $44.67 for appointment fees, $605.67 for prescription medication, $460.29 for hospitalization, $71.03 for auxiliary examination, $35.64 for transportation, $10.39 for special equipment, and $719.50 for formal care. The total cost is closely related to surgical treatment, dopamine agonist, and levodopa costs.

**Conclusion:**

The cost of PD patients in China is considerable and exceeds average economic capacity, especially antiparkinson medication and caring costs. This study may provide a reference for PD healthcare optimization in the future.

## 1. Introduction 

Parkinson's Disease (PD) is the second most prevalent neurodegenerative disorder after Alzheimer disease in the world [1, 2]. According to official data from the World Health Organization, the number of patients with PD has reached around 4 million in 2009 [3]. In Europe, the prevalence rate of PD is approximately 65.60–12,500 cases per 100,000 people and the annual incidence is about 5–346 cases per 100,000 people [4]. As the proportion of older people in the Chinese population has grown, the incidence of PD has also increased. An epidemiological survey indicated that the prevalence of PD is approximately 1.70% of the population aged over 65 years in China [5].

PD is characterized by four major symptoms: resting tremors, rigidity, bradykinesia, and postural instability [6]. Furthermore, patients with PD experience a diverse range of nonmotor symptoms; cognitive dysfunction; urinary complications; autonomic impairments, such as hypotension, constipation, and insomnia; neuropsychiatric symptoms, such as depression, anxiety, hallucinations, apathy, and compulsive disorder; and sensory disorders, such as numbness, pain, and smell disorder. These symptoms reflect the multisystem nature of the disorder and result in poor quality of life for affected patients [7].

Given the growing incidence of PD in China, expenditures for PD treatment and hospital care are beginning to exert a heavy economic burden on both patients' families and society. In comparison with literature in western countries on the medical costs and related social expenditure of PD patients [2, 8–11], little research on the topic has been done in China. In this study, a professional survey was prepared and administered to PD patients in China. This was followed by a systematic analysis of the main PD costs and contribution to total expenditure in China.

## 2. Design and Methods

### 2.1. Participants

A total of 116 participants with PD were recruited from the Movement Disorder Clinic of Tianjin Union Medical Center between August 2015 and November 2015. The study was approved by the Ethics Committee of Tianjin Union Medical Center. A declaration of informed consent was signed by each participant. All patients were diagnosed by the senior neurologists specializing in movement disorders and fulfilled the UK Brain Bank criteria for idiopathic PD [12]. The severity of PD was assessed following the Unified Parkinson's Disease Rating Scale [13] and the Hoehn and Yahr (HY) scale [14].

### 2.2. Survey Design

Telephone and questionnaire surveys were applied to both patients and MedCare suppliers to obtain all participants' health information and PD-related costs during the 12-month study period (2015 year). For those participants with symptoms of dementia and language dysfunction, additional information was obtained from MedCare suppliers. The costs of treating nonmotor symptoms were excluded from this research.

### 2.3. Direct Cost

Direct costs include the costs for rehabilitation, outpatient care, surgery, special equipment, auxiliary examination, transportation, and medications, such as dopaminergic drugs, catechol-O-methyl transferase inhibitors, monoamine oxidase B inhibitors, anticholinergic, and amantadine. Official standard prices for each item were used in all cost calculations. All costs were converted into $.

### 2.4. Formal Care

Some PD patients receive care from professional nurse workers at home or in a sanitarium. This is defined as formal care. Other patients receive home care from family members, friends, or relatives. This is defined as informal care. Costs for informal care were not included in this study.

The cost for transportation to seek medical treatment or advice includes payment for bus, taxi, subway, or gas and was calculated based on local transportation fees.

### 2.5. Indirect Cost

Indirect cost was assessed according to the loss of income caused by premature retirement due to PD. The official retirement age is 60 for males and 55 for females in China. The indirect cost per year was calculated as the difference between the current monthly pension for early retirement and monthly payment before retirement multiplied by 12.

### 2.6. Statistical Analysis

The data were analyzed using SPSS 22.0 software. The rank-sum test was used for univariate analysis to identify the cost-driving factors. Multiple linear regression was used to analyze independent cost-driving factors. All data were presented with 95% confidence intervals. A *P* value < 0.05 was considered to indicate statistical significance.

## 3. Results

### 3.1. Clinical Parameters and Demographic Characteristics of the Participants

One hundred and sixteen PD patients (72 males and 44 females) were enrolled in this study. There were 27 patients (23.28%) aged ≤ 60 years, 48 patients (41.38%) aged 61–70 years, and 41 patients (35.34%) aged ≥ 71 years. The mean duration of PD was 7.3 years (range 0.5–23 years), and the severity of PD was mostly in the range of HY stages I–III (Table 1). Among the 116 participants, 3 (2.6%) received surgical therapy and 113 (97.4%) received conservative treatment with medications. The detailed information of the 116 participants is shown in Table 1.

### 3.2. Cost Calculation

According to our survey and analysis of the 116 PD participants in China, the average annual cost for PD treatment was $3,225.94. The total cost includes direct and indirect costs. The direct cost is much higher than the indirect cost (77.60% versus 22.40%). The detailed expense information is shown in Table 2.

### 3.3. Direct Cost

The average direct cost was $2,503.46 annually. Of this amount, surgery fees accounted for 17.24% of total costs, antiparkinsonian drugs accounted for 18.77%, formal care accounted for 22.30%, inpatient care accounted for 14.27%, and other expenditures, including auxiliary, transportation, appointment fee, and special equipment, accounted for 5.01%.

**Figure 1:**
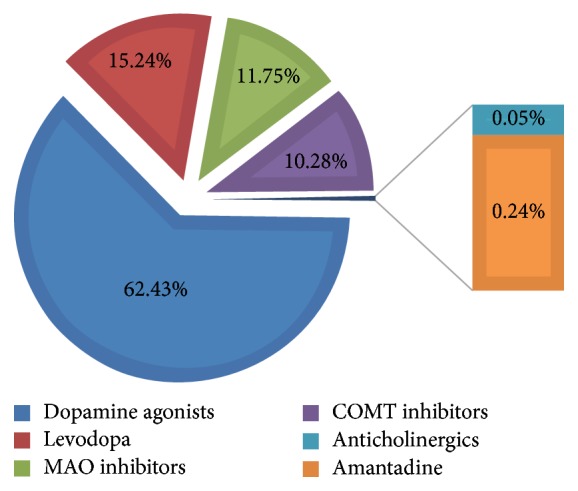
Direct costs of antiparkinsonian medicine.

The antiparkinsonian medication cost depended on the drug type(s) used. The medication expense details are shown in Table 2 and Figure 1. Notably, dopamine agonists accounted for 62.43% of expenditures on antiparkinsonian medicine. Among the 116 participants in our study, levodopa was used by 88.79% of patients, Dopamine agonists by 67.24%, Catechol-O-methyl transferase (COMT) inhibitor by 8.62%, Amantadine by 28.45%, Monoamine oxidase B (MAO-B) inhibitor by 16.38%, and Anticholinergics by 16.38%. Levodopa is the main antiparkinsonian medicine widely used by patients with PD in China.

### 3.4. Indirect Cost

Among the 116 participants, 22 patients had retired early because of PD. The average annual indirect cost was $722.48. Notably, the indirect cost was higher for patients with a disease duration of 6–10 years compared with that of those with a duration of ≤5 years and >10 years.

### 3.5. Hospitalization Cost

Among the 116 participants, 24.14% had received hospitalized treatment in the department of neurology. In this study, the average annual hospitalization cost per patient was $460.29, including PD-related examinations, nursing, medication, and transfusion treatment (Table 2).

### 3.6. Surgery Costs

Emerging evidence indicates that deep brain stimulation (DBS) surgery treatment can improve life quality and reduce both medication costs and motor complications among patients with PD; however, the surgery is expensive [15, 16]. The average annual cost of surgery for each person is approximately $556.27.

### 3.7. Cost-Driving Factors

Univariate analyses were used to analyze the correlation between factors related to PD and the total cost. As shown in Table 3, sex, age, delayed diagnosis, and patient history did not show significant correlation with the total cost. However, HY stage, duration of PD, surgical therapy, levodopa, and dopamine agonist were found to have positive correlations with the total cost (*P* < 0.05). The relationships between the independent cost-driving factors were evaluated by multivariate analyses (Table 4).

## 4. Discussion

The costs of PD in western countries have been studied and findings indicate that the disease is a serious economic burden in those countries as well. The average PD-related annual cost per patient was $12,215 in the Czech Republic in 2004 [17], $5,808 in Russia [18], $13,367 in Italy [3], $22,800 in the United States [2], and $36,085 in the UK [19]. Herein, we calculated the PD-related annual cost in China, which was $3225.94 per patient in 2015. Although the cost is lower in China than that in western countries, it still exerts a heavy economic burden due to the low level of average income in China.

From our study, antiparkinsonian medicine is responsible for 24.19% of direct cost for patients with PD in China, which is very close to the worldwide range of 22.00–58.00% [8, 10, 15, 20, 21]. The cost for prescription drugs in European countries is higher than that in China, mainly due to the usage proportion of levodopa, which is 97.20% in Germany, for example, and 88.79% in China [8]. Levodopa is one of the most common medicines for PD treatment in both developed and developing countries, and it is generally known to have a “honeymoon period,” after which its effectiveness is reduced and the patient experiences a variety of motor complications in the advanced stages of the disease. For early onset and advanced PD patients, dopamine agonists are also widely used for disease therapy. Therefore, dopamine agonists costs are an independent cost-driving factor. COMT and MAO-B inhibitors are used less often by patients in our study compared with patients in Germany (8.62% versus 23.40% for COMT and 16.38% versus 26.90% for MAO-B, resp.) [8]. The higher cost of these two agents in China likely explains this difference.

In comparison with the ~27.59% of total cost paid for formal care in Czech [17], formal care accounts for only 22.30% of total costs in China according to the present study. Formal care provided by a home nurse or professional caring service is very common in developed countries. However, formal care at home is not covered by health insurance in China, and patients have to pay for such services using personal means. Therefore, in-home care is usually provided by family members or relatives to reduce PD expenditures in China.

In addition, our study indicated a considerable difference between the indirect costs for patients with PD in developed countries and that of patients in China. The indirect cost is approximately $5200 (41.94% of total cost) in Europe [20]. However, it is only $722.48 (22.40% of total cost) in China. The difference is mainly due to the lower average salary and family income in China. For farmers in China, the family income decreases when a family member develops PD, especially in the advanced stage of the disease. For employees, the monthly income from a retirement pension is usually far lower than the previous salary.

Surgical treatment is one of the independent cost-driving factors for PD patients (*P* < 0.05). Dams et al. reported that DBS treatment is a cost-effective option for PD patients [16]. Although PD patients usually do not receive DBS treatment in the early stage of the disease due to surgical complications, potential risks, and additional costs as described by Kim et al. [22], several clinical trials demonstrated that DBS can significantly relieve motor complication and symptoms and improve patients' quality of life [23–25]. In our study, the majority of PD patients in HY stages III and IV did not opt for surgical therapy owing to the high expense.

Surgical treatment, dopamine agonist, and levodopa costs have been identified as three independent cost-driving factors of PD in the present study. HY stage has been confirmed as the main cost-driving factor [24, 26, 27]; however, our result is not consistent with findings in the literature. With the aggravation of the disease, patients in HY stages IV and V accounted for a small proportion of the study sample (15.52%).

In conclusion, although the total cost of PD in China is less than that in western countries, it represents a heavy economic burden for patients, their families, and society due to lower average family income and economic conditions in China. This study may provide a reference for optimizing care for patients with PD and health insurance distribution in the future.

## Figures and Tables

**Table 1:** Demographic characteristics and clinical parameters of the 116 participants.

Characteristics		Total		Age group					
				≤60 years		61–70 years		≥71 years	
		*n*	100%	*n*	100%	*n*	100%	*n*	100%
Total		116.00	100.00%	27.00	23.28%	48.00	41.38%	41.00	35.34%
Sex									
Male		72.00	62.07%	19.00	16.38%	29.00	25.00%	24.00	20.69%
Female		44.00	37.93%	8.00	6.89%	19.00	16.38%	17.00	14.66%
HY stage									
HY I		32.00	27.59%	10.00	8.62%	15.00	12.93%	7.00	6.03%
HY II		37.00	31.89%	8.00	6.90%	17.00	14.66%	12.00	10.34%
HY III		29.00	25.00%	6.00	5.17%	11.00	9.48%	12.00	10.34%
HY IV		9.00	7.76%	2.00	1.72%	3.00	2.59%	4.00	3.45%
HY V		9.00	7.76%	1.00	0.86%	2.00	1.72%	6.00	5.17%
Duration of disease									
1–5 years		71.00	61.21%	17.00	14.66%	29.00	25.00%	25.00	21.55%
6–10 years		33.00	28.45%	8.00	6.92%	12.00	10.34%	13.00	11.21%
>10 years		12.00	10.34%	2.00	1.72%	7.00	6.03%	3.00	2.59%
Past history									
Hypertension		8.00	6.90%	2.00	1.72%	2.00	1.72%	4.00	3.45%
Diabetes		4.00	3.45%	1.00	0.86%	3.00	2.59%	0.00	0.00%
Angiocardiopathy		11.00	9.48%	0.00	0.00%	4.00	3.45%	7.00	6.03%
CI		7.00	6.03%	0.00	0.00%	3.00	2.59%	4.00	3.45%
No		86.00	74.14%	24.00	20.69%	36.00	31.03%	26.00	22.41%
Delayed diagnosis									
≤2 years		91.00	78.45%	21.00	18.10%	39.00	33.62%	31.00	26.72%
≤5 years		18.00	15.52%	5.00	4.31%	4.00	3.45%	9.00	7.76%
>5 years		7.00	6.03%	1.00	0.86%	5.00	4.31%	1.00	0.86%
Surgical therapy									
Yes		3.00	2.59%	1.00	0.86%	0.00	0.00%	2.00	1.72%
No		113.00	97.41%	26.00	22.41%	48.00	41.38%	39.00	33.62%

**Table 2:** Annual PD expenses ($, 2016 values) of the 116 participants.

	≤5 years	6–10 years	≥11 years	Total
	Mean (95% CI)	Mean (95% CI)	Mean (95% CI)	Mean (95% CI)
Direct costs	1837.41 (588.00–3086.81)	3244.01 (1623.48–4864.54)	4407.80 (2919.82–5895.78)	2503.46 (1600.49–3406.44)
Direct medical costs	1546.68 (402.56–2690.80)	1872.53 (615.89–3129.18)	2499.30 (1576.99–3421.61)	1737.93 (957.50–2518.35)
Antiparkinsonian drug	593.46 (490.13–696.79)	615.59 (385.67–845.50)	650.65 (440.45–860.86)	605.67 (514.86–696.48)
Levodopa (*n* = 103)	100.90 (84.38–117.42)	111.00 (92.61–129.39)	99.47 (59.80–139.14)	92.32 (80.42–104.21)
Dopamine agonists (*n* = 78)	613.69 (527.64–699.73)	521.86 (404.62–639.10)	405.79 (281.81–529.77)	378.11 (313.87–442.34)
Anticholinergics (*n* = 19)	2.26 (1.51–2.99)	1.50 (0.81–2.19)	1.40 (−0.34–3.14)	0.30 (0.16–0.44)
Amantadine (*n* = 33)	5.30 (4.38–6.22)	5.14 (4.09–6.19)	4.67	1.47 (1.01–1.94)
MAO-B inhibitors (*n* = 19)	527.67 (365.50–689.84)	376.27 (212.58–539.96)	312.60	71.18 (38.48–103.88)
COMT inhibitors (*n* = 10)	643.85	840.59 (214.49–1466.68)	643.85	62.29 (22.70–101.87)
Inpatient care (*n* = 28)	1679.48 (1503.60–1855.35)	1679.48 (1503.60–1855.35)	1679.48 (1503.60–1855.35)	460.29 (295.67–624.90)
Appointment fee (*n* = 116)	43.24 (41.83–44.65)	44.47 (43.55–45.40)	53.66 (42.58–64.74)	44.67 (43.22–46.12)
Surgery care (*n* = 3)	38551.15	12987.85 (−17066.46–43042.17)	0	556.27 (−171.12–1283.65)
Auxiliary inspections costs (*n* = 50)	164.85 (163.46–166.24)	164.16	164.16	71.03 (55.95–86.11)
Direct nonmedical costs	290.72 (65.97–515.48)	1371.48 (726.01–2016.96)	1908.50 (812.35–3004.66)	765.54 (496.72–1034.36)
Special equipment (*n* = 26)	46.37	46.37	46.37	10.39 (6.82–13.96)
Transportation fee (*n* = 116)	34.64 (23.55–45.73)	34.04 (25.64–42.43)	45.98 (29.12–62.85)	35.64 (28.35–42.94)
Formal care (*n* = 26)	2937.60 (1224.90–4650.30)	3362.95 (2688.08–4037.82)	3159.77 (2434.92–3884.62)	719.50 (453.48–985.53)
Indirect costs	3386.88 (2373.67–4400.09)	4068.65 (2977.96–5159.35)	3686.40 (2649.94–4722.86)	722.48 (428.25–1016.71)
Productivity losses (*n* = 22)	3386.88 (2373.67–4400.09)	4068.65 (2977.96–5159.35)	3686.40 (2649.94–4722.86)	722.48 (428.25–1016.71)

**Table 3:** Univariate analyses of contributions of various factors to the cost of PD ($, 2016 values).

Characteristics	*N*	Mean	Median	*P*_25_	*P*_75_	Mean rank	*P*
Sex							0.285^*∗*^
Male	72	2626.44	1141.15	587.03	3461.17	8.05	
Female	44	4206.96	1373.20	758.55	4491.21	9.04	
Age							0.291^*∗∗*^
≤60 years	27	4193.49	1583.65	747.62	6880.35	9.55	
61–70 years	48	2280.62	890.48	631.19	2413.64	7.73	
≥71 years	41	3695.51	6295.98	513.73	4054.51	8.50	
Duration of disease							0.000^*∗∗*^
1–5 years	71	2075.92	796.02	536.57	1583.65	6.98	
6–10 years	33	4600.23	2774.03	683.60	6006.25	9.75	
>10 years	12	6251.00	6209.40	1908.07	9664.85	13.31	
Delayed diagnosis							0.059^*∗∗*^
≤2 years	91	3152.85	978.64	627.20	2921.30	8.06	
≤5 years	18	2877.63	1208.53	584.61	5292.34	8.63	
>5 years	7	5071.96	2774.03	1822.18	9408.40	12.57	
HY stage							0.000^*∗∗*^
HY I	32	1055.23	749.72	425.56	1445.52	5.93	
HY II	37	1352.50	850.04	348.86	1480.91	6.72	
HY III	29	6917.09	3858.03	936.91	9293.66	11.14	
HY IV	9	6264.64	5379.19	1421.05	10206.00	12.78	
HY V	9	3713.63	4282.27	1541.82	5441.76	11.19	
Past history							0.163^*∗∗*^
No	86	2760.31	999.28	602.26	3902.76	8.11	
Hypertension	8	11643.62	5324.07	1012.57	18502.35	11.56	
Diabetes	4	1099.16	659.39	278.21	2359.87	5.26	
Angiocardiopathy	11	1778.42	1316.31	693.57	2723.36	8.51	
CI	7	2816.39	2687.50	958.62	4557.04	10.35	
Surgical therapy							0.000^*∗*^
No	113	2468.63	1252.39	635.19	3741.53	8.21	
Yes	3	31751.49	2578.24	21196.31		16.56	
Antiparkinsonian drug levodopa costs							0.046^*∗∗*^
≤455.625	41	2063.41	791.36	695.40	2023.34	7.51	
≤675	40	2675.48	1004.27	341.80	3587.03	7.73	
≤911.25	11	8073.97	4282.27	1019.93	8535.38	11.17	
>911.25	21	3907.39	2426.61	685.98	5657.59	9.89	
Dopamine agonists costs							0.000^*∗∗*^
0	38	1672.29	427.42	277.88	2276.65	5.84	
≤2365.2	10	8196.79	6410.02	484.27	13114.41	10.66	
≤3547.8	55	3350.52	1252.39	791.36	4122.04	9.21	
>3547.8	13	3416.75	1469.23	1469.23	4403.86	10.93	

^*∗*^Rank-sum test of two independent samples. ^*∗∗*^Rank-sum test of multigroup independent samples.

**Table 4:** Multivariate analyses of the cost-driving factors.

Model	Unstandardized coefficients		Standardized coefficients	*T*	Sig
	*B*	SE	Beta		
Constant	−25258.657	9804.132		−2.576	0.011
Surgical therapy	90937.539	8562.191	0.686	10.621	0.000
Levodopa costs	5996.662	2379.178	0.166	2.520	0.013
Dopamine agonists costs	5496.075	2583.129	0.144	2.128	0.036

The statistical method of Table 4 is multiple linear stepwise regression analysis.
